# Properdin and factor H production by human dendritic cells modulates their T‐cell stimulatory capacity and is regulated by IFN‐γ

**DOI:** 10.1002/eji.201646703

**Published:** 2017-03-13

**Authors:** Karen O. Dixon, Joseph O'Flynn, Ngaisah Klar‐Mohamad, Mohamed R. Daha, Cees van Kooten

**Affiliations:** ^1^ Department of NephrologyLeiden University Medical CenterLeidenThe Netherlands; ^2^ Evergrande Center for Immunologic Diseases at Harvard Medical School and Brigham and Women's HospitalBostonMAUSA; ^3^ Program in Cellular and Molecular MedicineBoston Children's HospitalHarvard Medical SchoolBostonMAUSA

**Keywords:** Complement, Cytokines, Factor H, IFN‐γ, IL‐27, Properdin, Tolerogenic dendritic cells

## Abstract

Dendritic cells (DCs) and complement are both key members of the innate and adaptive immune response. Recent experimental mouse models have shown that production of alternative pathway (AP) components by DCs strongly affects their ability to activate and regulate T‐cell responses. In this study we investigated the production and regulation of properdin (fP) and factor H (fH) both integral regulators of the AP, by DCs and tolerogenic DCs (tolDCs). Both fP and fH were produced by DCs, with significantly higher levels of both AP components produced by tolDCs. Upon activation with IFN‐γ both cells increased fH production, while simultaneously decreasing production of fP. IL‐27, a member of the IL‐12 family, increased fH, but production of fP remained unaffected. The functional capacity of fP and fH produced by DCs and tolDCs was confirmed by their ability to bind C3b. Inhibition of fH production by DCs resulted in a greater ability to induce allogenic CD4^+^ T‐cell proliferation. In contrast, inhibition of fP production led to a significantly reduced allostimulatory capacity. In summary, this study shows that production of fP and fH by DCs, differentially regulates their immunogenicity, and that the local cytokine environment can profoundly affect the production of fP and fH.

## Introduction

Dendritic cells (DCs) and the complement system are both integral members of the immune system participating in the first line of defence of the innate immune response and together further tailoring our adaptive immune response 1, 2, 3. The complement system is composed of three distinct pathways‐classical, lectin and alternative. Together these pathways serve as key mediators of the innate immune response, involved in defending the host from pathogens, removing immune complexes and facilitating efficient phagocytosis of apoptotic cells 2, 4. A key role of complement in the humoral arm of the adaptive immune response has been demonstrated through experimental depletion of complement, resulting in poorer antibody responses. Furthermore opsonization with complement fragments profoundly lowers the amount of antigen needed to induce antibody production 5, 6. Recently, there has been an increasing body of evidence demonstrating that complement may function more broadly as a link between innate and adaptive immunity. This includes murine studies which have demonstrated a role for the AP in the DC: T‐cell synapse 3, 7, 8, 9. Both APC and T‐cells have been shown to increase C3a and C5a receptor on their surface 10, while a surface regulator, decay accelerating factor, has been shown to be decreased, allowing for further complement activation 11, 12.

The AP is unique in that it can become auto activated via C3 hydrolysis generating a C3b like molecule which can bind covalently to an activating surface recruiting factor B, which is then cleaved by factor D generating a C3 convertase (C3bBb). This C3 convertase can convert further C3 to C3b, with stabilization of the enzyme by properdin (fP) allowing for propagation of the AP activation 13. Regulation of the AP is key, with fP promoting AP activation and negative regulators such as factor H (fH), limiting the availability of C3b, or acting as a cofactor for factor I, which degrades C3b. This delicate balance between activators and inhibitors of the AP is crucial for controlling complement activation 14. The majority of complement proteins are produced by the liver, although white blood cells including DCs can also act as a local source of certain complement proteins 15. The source of fP is largely restricted to white cells, including neutrophils 16, 17. fH is most abundantly expressed in the liver, but also here extra hepatic production has been described, which includes mesangial cells, endothelial cells, and fibroblasts 18, 19, 20, 21. This suggests that local cell populations, either resident or infiltrating, can significantly contribute to the overall complement activation at a particular site of inflammation.

DCs are distinct among other cells in the immune system as they are uniquely equipped to respond to a host of innate stimuli while also adapting and tailoring their functions in response to the local cytokine environment. This had led to the understanding that DCs are not just immunogenic but that tolerogenic DCs (tolDCs) also exist. These tolDCs are characterized by differences in cytokine production and expression of co‐stimulatory molecules, which has a direct impact on the strength and quality of their T‐cell stimulatory capacities 22, 23. Previous studies have demonstrated by RT‐PCR that DCs can express many components of the complement system 24, 25, however protein data to support this has been limited. Furthermore there are only limited data available on the regulation of AP component production by DCs or particularly tolDC populations. Local production of complement components such as C3 by APCs at the site of inflammation or immunological synapse can be instrumental in the immune response 8. With the demonstration that local AP activation at the interface of APC and T cells plays a key role in the regulation of T‐cell responses 26, regulation of the AP in the local environment will be key in controlling the strength of the T‐cell response. Therefore, we investigated the regulation of two integral modulating factors of the AP, fP, and fH. Combined both molecules have the ability to influence the balance of the AP toward activation or regulation.

In this study, we demonstrate that DCs are a source of fP and fH and that tolDCs are superior producers of both components. We observed a differential regulation of fP and fH, whereby IFN‐γ profoundly inhibited fP in both cell types, while simultaneously increasing production of fH. This distinct dual effect was found to be unique for type II IFN. IL‐27 is a member of the IL‐12 family, which demonstrates some functional homogeny with IFN‐γ in human dendritic cells and hepatocytes 27, 28, 29. In line with this functional homogeny with IFN‐γ, IL‐27 did significantly enhance fH levels in DC supernatants. Importantly, RNA interference of fH in DCs increased their allostimulatory capacity, while siRNA targeting of fP decreased T‐cell proliferation. Taken together, our data suggest that the local cellular and cytokine microenvironment are crucial for overall complement regulation and thereby T‐cell immunity.

## Results

### TolDCs display elevated levels of properdin and fH

**Figure 1:**
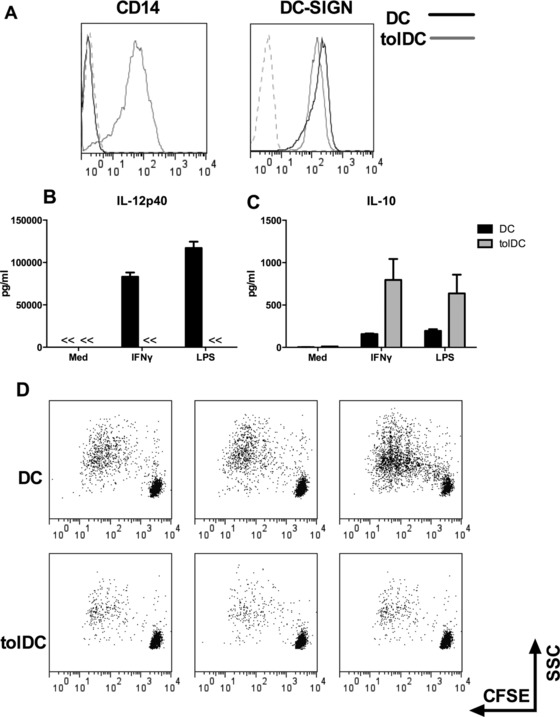
Characterization of DCs and tolDCs. Monocytes were isolated from PBMCs and DCs were generated by culturing in medium supplemented with GM‐CSF and IL‐4. TolDCs were generated by addition of dexamethasone (Dex) only at the start of culture (day 0). (A) Representative histograms of cell surface FACS analysis on DC and tolDC at day 6 of culture, stained for CD14 and DC‐SIGN. Histograms shown were gated on PI negative cells, dashed lines represent isotype control mAb staining. Results shown are from a representative experiment of three independent experiments performed with three different donor samples. Cells were stimulated with IFN‐γ or LPS and the supernatants were harvested and (B) IL‐12p40 and (C) IL‐10 was measured by ELISA. Data are shown as mean + SD and are pooled from four independent experiments performed with 4 different donor samples. (D) DCs were co‐cultured with CFSE labeled allogenic CD4^+^ T cells at a ratio of 1:10. On day 5 the T cells were harvested and the CFSE staining was analyzed using flow cytometry. Data shown is representative of three independent experiments performed with three different donor samples.

Monocyte derived DCs and tolDCs were generated as previously described 30, 31, 32. Flow cytometric analysis revealed that DCs display characteristic features, expressing high levels of DC‐SIGN but little or no expression of CD14. In contrast, although tolDC display similar levels of DC‐SIGN, they maintain their expression of CD14 upon differentiation from monocyte to DC (Fig. 1A). Both cell types were stimulated with IFN‐γ or LPS and assessed for IL‐12 and IL‐10 production. DCs produced significant levels of IL‐12 upon stimulation while IL‐10 was relatively low. In contrast tolDCs demonstrated a typical profile with absence of IL‐12 and high IL‐10 production (Fig. 1B and C). In addition these tolDCs had a poor ability to stimulate allogenic T‐cell proliferation (Fig. 1D).

**Figure 2:**
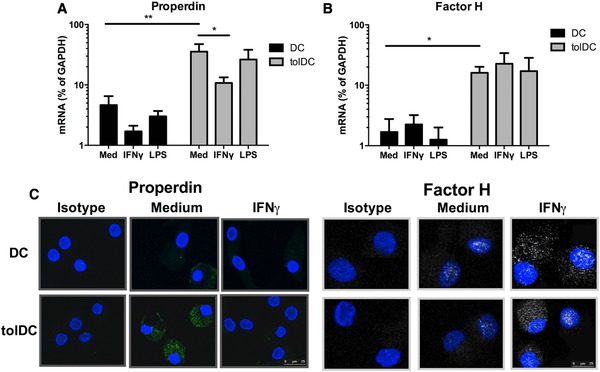
Expression and production of properdin and fH by human DCs and tolDCs. Dendritic cells were harvested after 6 days of culture, washed and stimulated with IFN‐γ or LPS, after which mRNA was isolated followed by cDNA synthesis. The transcript levels of (A) Properdin (*CFP*) (B) Factor H (*CFH*) were determined by RT‐PCR. GAPDH mRNA expression from the same samples was used as an endogenous reference gene (relative mRNA expression). Data are shown is mean + SD of 8 independent experiments performed with eight different donor samples.* one‐tailed *t*‐test *p*≤0.047,**p≤0.082 (C) DCs were cultured on 8‐well chamber slides and either untreated or stimulated with IFN‐γ followed by incubation with Brefeldin. The cells were then fixed and permeabilized, followed by incubation with anti‐fP or anti‐fH followed by detection with GaM‐Alexa^488^. Fluorecent images are from a single experiment representative of experiments performed with three different donor samples. Scale bar 25 μm.

To investigate the contribution of DCs to the production of alternative pathway related proteins we first assessed by RT‐PCR if *CFP* and *CFH* were expressed in both DC and tolDC populations. We demonstrate that DCs and tolDCs expressed both factors, and that tolDCs showed more than 10 fold higher transcription of both *CFP* and *CFH* compared to DCs (Fig. 2A and B). IFN‐γ stimulation of tolDCs resulted in lower mRNA expression of *CFP* as compared to the unstimulated state, although not statistically significant a similar trend was observed in DCs. In contrast, DCs and tolDCs stimulated with IFN‐γ or LPS demonstrated no significant overall change in *CFH* expression (Fig. 2A and B).

We assessed fP and fH protein production within DCs and tolDCs using intracellular microscopy. Using this method we could demonstrate expression of fP and fH protein in both DCs and tolDCs (Fig. 2C). Incubation with IFN‐γ reduced the presence of fP, which is in line with our findings at mRNA level. In contrast to fP, IFN‐γ appeared to increase the expression of fH.

### IFN‐γ exerts opposing effects on the production of fP and fH by DCs and tolDCs

**Figure 3:**
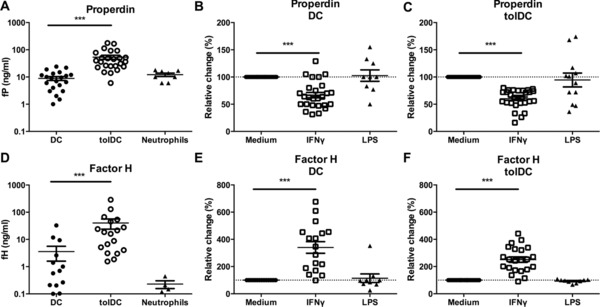
IFN‐γ exerts opposing effects on the production of fP and fH by DC and tolDC. DCs were harvested on day 6, and neutrophils were isolated from blood as described in the Materials and methods, followed by replating for another 24 h. (A) fP and (D) fH production was measured using ELISA. Data shown is mean ± SD of 8–24 (fP) or 4–18 (fH) independent experiments which were pooled together. Cells were stimulated for 24 h after which (B, C) fP and (E, F) fH was determined by ELISA. Levels upon stimulation were expressed as percentage change relative to unstimulated conditions of the same donor (relative change). Data shown is mean ± SD of 9–28 independent experiments which were pooled together, one‐tailed *t*‐test ***p≤0.0001.

To further assess the regulation of fP and fH protein, we performed ELISAs on cell culture supernatants. In line with Q‐PCR data (Fig. 2), we found that tolDCs produced significantly more fP and fH compared to DCs (Fig. 3A and D). For comparison, supernatants from neutrophils, a widely accepted source of fP, were shown to produce levels of fP similar to DCs, but with only minimal production of fH. To investigate whether the differential regulation of fP and fH was a general feature of mature DCs or was activation dependent, we compared stimulation with either IFN‐γ or LPS. In view of the large differences in fP and fH production (Fig. 3A and D), we calculated the relative change upon stimulation for each donor measured, with the immature/medium state set at 100%. In both DC and tolDC we observed that fP production was significantly reduced in the presence of IFN‐γ (Fig. 3B and C). Regulation of fH demonstrated the opposite effect, where both cell types showed a significant increase in fH production upon treatment with IFN‐γ (Fig. 3E and F). Despite the strong induction of cytokine production (Fig. 1), LPS did not show a significant effect on the production of either fP or fH.

### Type I IFNs do not possess the opposing properties of IFN‐γ, while IL‐27 specifically increases fH

**Figure 4:**
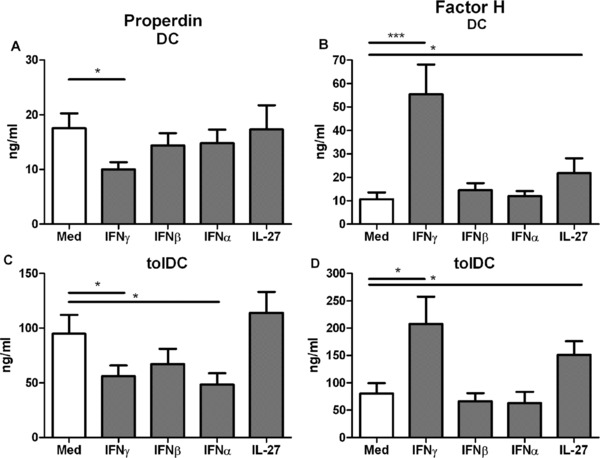
Type I IFNs decrease fP production but do not increase fH, while IL‐27 specifically increases fH. Dendritic cells were harvested after 6 days of culture and stimulated with IFN‐γ, IFN‐β, IFN‐α, or IL‐27. Supernatants were harvested after 48 h the and (A, C) fP and (B, D) fH was measured by sandwich ELISA. Data shown is the mean ±SD of 8–13 independent experiments pooled together, one‐tailed *t*‐test *p≤0.04, ***p≤0.0016.

We addressed whether other members of the Interferon family, or IL‐27, could also possess this dual role in regulating alternative pathway components in DCs. Similar to IFN‐γ stimulation IFN‐α, but not IFN‐β, did significantly reduce fP levels in tolDC supernatants. No regulation of fP was observed in DC treated with type I IFN (Fig. 4A and C). In contrast, IL‐27 did not demonstrate any clear ability to regulate fP production. The levels of fH remained unchanged in both DC and tolDCs stimulated with either IFN‐α or IFN‐β but were significantly upregulated upon IL‐27 and IFN‐γ stimulation respectively (Fig. 4B and D).

### DC‐derived fP and fH exhibit traditional functional characteristics

**Figure 5:**
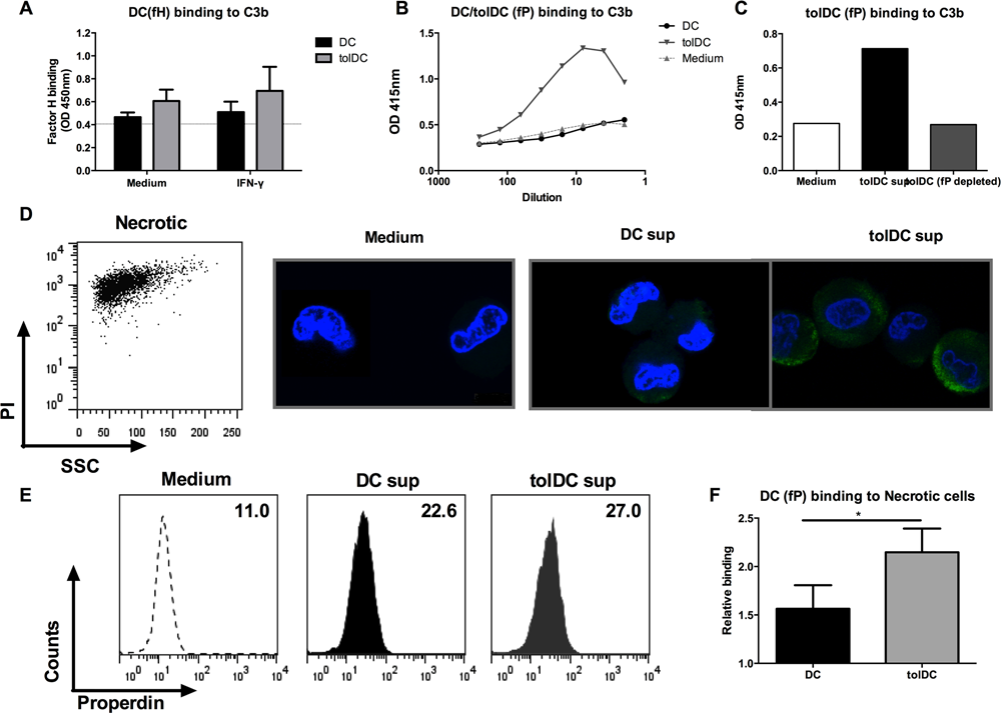
DC derived fP and fH exhibit traditional functional characteristics. Dendritic cells were harvested after 6 days of culture and either untreated or stimulated with IFN‐γ. After 48 h the supernatants were harvested and incubated on ELISA plate coated with C3b, followed by detection of (A) fH and (B) fP. (C) To determine the specificity of fP binding the same C3b binding assay was performed with tolDCs supernatants either untreated or depleted of fP using pre‐coupled anti‐properdin beads. Data shown is representative of three independent experiments performed with three different donors. (D) Necrotic cells were generated and incubated with 48‐h supernatants from immature DC/tolDC, followed by detection of fP binding by fluorescent microscopy. Scale bars 25 μm. (F) The same experiment was performed followed by detection of fP binding by flow cytometry. Relative binding refers to DC/tolDC conditioned medium relative cell free medium where cell free medium was set as 1. Data shown is the mean ±SD of three independent experiments pooled together, one‐tailed *t*‐test *p≤0.0357.

We assessed whether fH found in DC supernatants can bind to C3b, a key characteristic of serum derived fH. Although we found minimal binding using supernatants of immature DC, the binding was enhanced in the supernatants from IFN‐γ‐stimulated DCs. This binding was more pronounced in supernatants of IFN‐γ‐stimulated tolDCs (Fig. 5A). We assessed the ability of fP derived from DCs and tolDCs to bind its traditional ligand C3b and found that while the amount produced by DCs might be too little for detection, fP derived from tolDCs showed a strong and dose‐dependent binding to C3b (Fig. 5B). To confirm the specificity of this assay we show that binding is inhibited upon depletion of fP using pre‐coupled anti‐properdin beads (Fig. 5C).

Recently it has been shown that fP possesses additional functions aside from C3b binding, and may act as an independent recognition molecule for necrotic and apoptotic cells 33. Necrotic cells were generated as previously described 30, and incubated with conditioned supernatant of DC or tolDC, followed by detection of fP binding. Using fluorescent microscopy, fP binding was detected on necrotic cells exposed to DC supernatant and this binding was even more evident on necrotic cells exposed to tolDC supernatants (Fig. 5D). This binding to necrotic cells was confirmed by flow cytometric analysis (Fig. 5E), and quantification across multiple donors showed that supernatants of tolDC demonstrated significantly higher levels of fP binding to necrotic cells compared to supernatant of DCs (Fig. 5F).

### Inhibition of fP in DC reduces T‐cell proliferation, while inhibition of fH enhances proliferation

**Figure 6:**
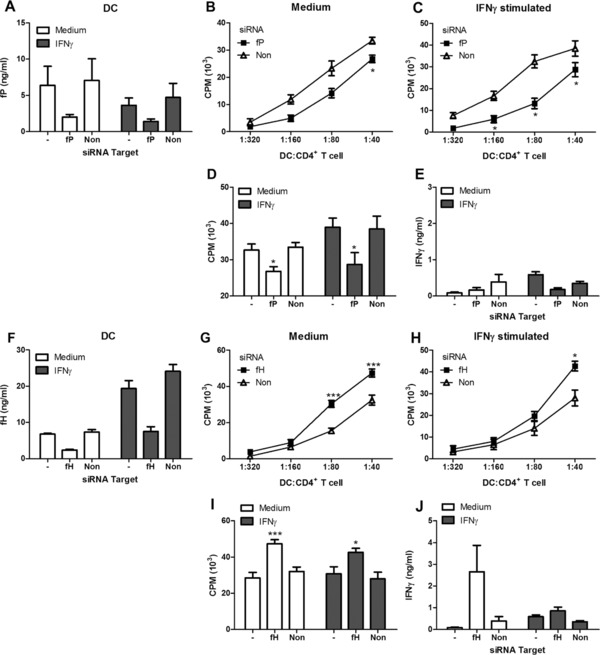
Inhibition of fP in DCs decreases the allostimulatory capacity of DCs while inhibition of fH enhances allogenic T‐cell proliferation. DCs were either non treated, or treated with siRNA targeting fP or non‐specific (non) target. After 24 h of culture, with or without IFN‐γ activation, supernatants were harvested and fP was measured by ELISA (A). DCs with either fP or control silencing, without (B) or with IFN‐γ (C) stimulation were co‐cultured with allogenic CD4^+^ T cells at indicated ratios. T‐cell proliferation was determined at day 5 of culture by ^3^[H] incorporation. Data shown are the mean ±SD of triplicate cultures. The mean ±SD of proliferation (D) and IFN‐γ production (E) at the 1:40 ratio of three independent experiments of cells silenced for fP is shown. DCs were either non treated, or treated with siRNA targeting fH or non‐specific (non) target. After 24 h of culture, with or without IFN‐γ activation, supernatants were harvested and fH was measured by ELISA (F). DCs with either fH or control silencing, without (G) or with IFN‐γ (H) stimulation were co‐cultured with allogenic CD4^+^ T cells at indicated ratios. T‐cell proliferation was determined at day 5 of culture by ^3^[H] incorporation. Data shown are the mean ±SD of triplicate cultures. The mean ±SD of proliferation (I) and IFN‐γ production (J) at the 1:40 ratio of three independent experiments of cells silenced for fH is shown. Data shown is the mean ±SD of 3 independent experiments pooled together, one‐tailed *t*‐test *p≤0.02, ***p≤0.0001.

In light of the recent evidence that AP components may play a role in the allostimulatory capacity of DCs 8, 34, we investigated whether silencing of fP or fH in human DCs could alter T‐cell activation in an allogenic setting. We successfully and specifically silenced fP and fH in both immature and IFN‐γ‐stimulated DCs by up to 60% on average (Fig. 6A and F). Cells treated with siRNA were harvested and co cultured with allogenic total CD4^+^ T‐cells at indicated ratios for 5 days. Proliferation was determined by ^3^[H]‐thymidine incorporation. We observed that silencing of fP in DCs significantly hampered their ability to induce allogenic T‐cell proliferation compared with DCs with non‐targeted siRNA (Fig. 6B). This inhibition of proliferation was even more pronounced when fP silencing was combined with IFN‐γ stimulation (Fig. 6C). The silencing procedure by itself did not affect the T‐cell stimulatory capacity and the reduced proliferation upon fP silencing was significant for different individual experiments (Fig. 6D). In these co‐cultures, IFN‐γ production was relatively low, but showed a trend for reduced production in IFN‐γ‐activated, fP‐silenced DC (Fig. 6E).

Similar experiments were performed using DCs treated with siRNA targeting fH. These cells induced significantly more T‐cell proliferation compared to non‐target (non) treated DCs (Fig. 6G). This was also observed, but to a lesser degree, with DCs activated with IFN‐γ (Fig. 6H). Both immature and IFN‐γ‐stimulated DCs silenced for fH exhibited more T‐cell proliferation, in particular at a ratio of 1:40 (Fig. 6I). We assessed the production of IFN‐γ in T‐cell supernatants and found that immature DCs silenced for fH induced more IFN‐γ production. In line with our proliferation data the stimulatory effect of silencing fH was less potent when the DCs were first activated with IFN‐γ (Fig. 6J). Taken together loss of fH induced more T‐cell proliferation while loss of fP decreased T‐cell proliferation, suggesting that the local complement activation is important in regulating DC: T‐cell responses.

## Discussion

Dendritic cells are a heterogeneous population of professional antigen presenting cells responsible for both the initiation of immunity and immunological tolerance. Recent murine studies have suggested that complement activation is a key determinant in the ability of APCs to direct T‐cell responses. In this study we show that production of fP and fH by DCs is subject to regulation by IFN‐γ and IL‐27 and that modulation of these regulators of the AP determines the potency of the T‐cell response. This implicates the local cellular and cytokine microenvironment as crucial mediators of overall complement regulation and subsequent T‐cell activation.

Several exciting studies over the last decade have introduced complement as a system that actively plays a role in regulating T‐cell immunity 3, 35. By generating bone marrow APCs from C3^−/−^ 9, 36, factor B^−/−^, factor D^−/−^, C3aR^−/−^ 37 and C5aR^−/−^ 38 mice, various groups demonstrated that DCs require local complement activation in order to become fully competent APCs. To date these studies have mainly focused on rodent models, however DCs from C3 deficient individuals 39 were also shown to have impaired DC differentiation. More recently, a role of C3a and C5a derived from human DC in T‐cell proliferation has been demonstrated in vitro and in a humanized mouse model 8. Further work on human DCs and the contribution of other complement factors has remained finite with mostly transcriptional analysis and limited protein data.

We focused on monocyte‐derived DCs and tolerogenic DCs and showed that both cells produced fP and fH in an immature state with tolDCs expressing much higher levels of both components. Some studies have demonstrated gene expression of fP and fH in plasmacytoid DCs 24. Although we did not perform PCR analysis on pDCs, we did test cellular supernatants and did not find any detectable levels of both components analysed in this study (data not shown).

Several complement components have been shown to possess GAS and ISRE elements in their promoters 40, 41, so we assessed the ability of IFN‐γ to regulate fP and fH. Interestingly, IFN‐γ possessed opposing roles by decreasing fP production, while increasing fH. Decrease of fP upon IFN‐γ stimulation has been observed in primary monocytes and THP‐1 cell lines 42, but to our knowledge this is the first report of this dual effect on fH and fP in human DCs. IFN‐γ is the only member of the type II IFNs, so we questioned whether the regulation of fP and fH was unique for this specific member, or was a broader feature of the IFN family, including type I IFNs. IFN‐α and IFN‐β behaved comparably in terms of fP and fH regulation and did not possess the same regulatory properties of IFN‐γ.

The IL‐12 family has been gaining attention as a key regulator of immunity and immunological tolerance 43, 44, and one family member, IL‐27 has been shown to exert some functions typically seen with IFN‐γ stimulation 27, 28, 45, 46. Interestingly IL‐27 stimulation of DCs did increase production of fH but did not influence fP production again stressing the uniqueness of IFN‐γ in its differential regulation of these two opposing molecules of the complement system.

Functionally both fP and fH were active in the traditional complement assessment by binding to C3b. In recent years fP has been shown to act as a recognition molecule 13, 33, 47 with the ability to bind to, among others, apoptotic 48 and necrotic cells 49. We found that fP from DCs could bind necrotic cells though we did not test whether this was independent of complement activation.

We have shown that cytokines can profoundly and differentially regulate the opposing regulators of the AP, fP, and fH. IFN‐γ stimulation of DCs also changes many phenotypical features of DCs including up regulating co‐stimulatory molecules such as CD80 and CD86. In order to establish whether simply shifting the balance of production of fP and fH could alter the allostimulatory capabilities of DCs we silenced fP or fH in DCs and co‐cultured these cells with allogenic CD4^+^ T‐cells. We found that inhibition of fH led to a significant increase in T‐cell proliferation while inhibition of fP led to decreased proliferation. DCs silenced for fP and activated with IFN‐γ were even further hampered in their allostimulatory capacity, likely because of the combined silenced by siRNA and decreased fP production by IFN‐γ.

The observation made with the local production and regulation of soluble regulators of complement activation are in line with the results obtained with the role of DAF in local C3 regulation and its role in regulating T‐cell responses 11. DAF is a membrane bound regulator, expressed both on T cells and APC, and presence of DAF results in a reduced half‐life of the C3 convertase. This activity is similar to the activity of fH, and both will result in a reduced C3 activation. The model explaining the role of DAF, has shown that a transient decrease of DAF on both T‐cells and APC is required to allow efficient T‐cell activation 7, 11. We confirmed that DAF is expressed on human DC and found that expression is higher on tolDC. However, at variance with fH, the expression of DAF was not affected by IFN‐γ stimulation (data not shown). Whereas both DAF and fH are increased on tolDC, it is an oversimplification to directly link this to the tolerogenic potential of these cells. First of all, tolDC also express increased amounts of properdin, a positive regulator of the C3 activation which prolongs the half‐life of the convertase, and therefore the net results remains to be determined. Moreover, tolDC display a strongly increased expression of tolerogenic molecules (IL‐10, B7‐H1) with simultaneous reduction of immunogenic factors (IL‐12, CD80/CD86). Further work is needed to fully establish whether the diverging T‐cell proliferation is a direct effect of fP and fH on the T‐cells or a consequence of altered local complement activation. Interestingly it has recently been demonstrated that exogenous fH can generate a regulatory state in DCs 50. In line with these findings we have observed inhibition of T‐cell proliferation with increasing concentrations of fH (Supporting Information Fig 2.), therefore it will be of special interest to further investigate the effect on differentiation of functional T helper subsets, in line with data in mice concerning the role of C3a and C5a signalling in T helper differentiation 26, 34.

Taken together our results demonstrate a novel role for fP and fH production by DCs to be a key regulator of T‐cell activation. Both molecules can be influenced by IFN‐γ whereas only fH is increased by IL‐27, indicating that the local cellular and cytokine environment can potentially influence the local complement activation and in turn local T‐cell responses.

## Materials and methods

### Cell culture and reagents

Human monocytes were isolated from buffy coats obtained from healthy donors using Ficoll density gradient centrifugation followed by positive selection using anti‐CD14 MACS microbeads (Miltenyi Biotech GmBH, Bergisch Gladbach, Germany). DCs were generated and cultured in RPMI 1640 supplemented with 10% heat inactivated fetal calf serum (FCS), 90 U/mL penicillin, and 90 μg/mL streptomycin (Gibco/Life technologies, Breda, The Netherlands), 5 ng/mL GM‐CSF and 10 ng/mL IL‐4 (Biosource Europe, Belgium), as described before. Tolerogenic DC (tolDCs) were generated by addition of Dexamethasone (10^−6^M Dex) (Pharmacy, L.U.M.C., Leiden, the Netherlands) only at the start of culture (day 0) 32. Cultures were refreshed with medium containing cytokines on day 3. For stimulation experiments, immature DCs were harvested on day 6, washed, and seeded accordingly, followed by addition of 200 ng/mL LPS (*E.Coli EH100* Enzo, Belgium), 100 ng/mL each of IFN‐γ, IFN‐α, IFN‐β (Peprotech) or 100 ng/mL IL‐27 (R&D). Neutrophils were isolated as previously described 51. Briefly, blood from healthy donors was collected using ACD tubes (BD Vacutainer) and neutrophils were isolated by Ficoll‐Paque and Dextran T‐500 gradients (Sigma Aldrich). The preparation contained greater than 90% neutrophils as confirmed by flow cytometry using CD16 (R&D Systems), CD11b (BD Biosciences), and CD66b (AbD Serotec) antibodies.

### Flow cytometry

For cell surface flow cytometric analysis, cells were harvested, washed, and stained for 30 min at 4°C in FACS buffer (PBS, 0.5% heat inactivated NHS, 1% BSA, 0.02% NaN_3_) with anti‐CD14 MΦ P9 (BD Biosciences, San Diego, CA, USA) or anti‐DC‐SIGN (R&D Systems, Wiesbaden, Germany). Non‐conjugated antibodies were detected with PE‐conjugated goat‐anti‐mouse Ig (Dako, Glostrup, Denmark). Isotype matched control antibodies were used to determine the level of background staining. The fluorescence was measured on an FACS Calibur flow cytometer, and data were analyzed with Cell Quest Software (BD Biosciences, San Diego, CA, USA) and FlowJo Software (Tree Star, USA).

### mRNA isolation, cDNA synthesis, and RT‐PCR

Cells were harvested and mRNA was isolated from DCs using an Rneasy kit according to the manufacturer's instructions (Qiagen, Hilden, Germany). DNA was digested using the on‐column RNase‐free DNase set. cDNA was synthesized using a reverse transcription system kit (Promega) following the manufacturer's guidelines and stored at −20°C until analysis. Specific primers for human Properdin (*CFP*), Factor H (*CFH*), and GAPDH (Table 1) were designed using the computer software Oligo explorer and synthesized at Biolegio. Primer specificity was tested by homology search with the human genome (basic local alignment search tool or BLAST; National Center for Biotechnology Information) and later confirmed by electrophoresis through 2% agarose gels containing ethidium bromide followed by visualization under UV light. GAPDH was used as an endogenous reference gene. For each sample, the relative abundance of target mRNA was calculated from the obtained C_t_ values for the target gene and expressed relative to the endogenous reference gene GAPDH. A table demonstrating the range of CT values for fP and fH expression is included (Supporting Information Table 1).

**Table 1:** Real‐time PCR oligonucleotide sequences

Gene	NCBI ID	Protein ID	Forward sequence	Reverse sequence
*CFP*	5199	Proper din	GTAATCACCCTGCTCCCAAG	TTGCGGCTTCGTGTCTCC
*CFH*	3075	fH	GCACACAAGATGGATGGTCG	GGTCTGCGCTTTTGGAAGAG
*GAPDH*	2597	GAPDH	TTCCAGGAGCGAGATCCCT	CACCCATGACGAACATGGG

### DC‐T‐cell co culture

Allogeneic CD4^+^ T‐cells were isolated from buffy coats by negative selection using the MACS CD4^+^ T‐Cell Isolation Kit II (Miltenyi Biotec). DCs from all conditions were harvested after 24 h, washed, and plated in 96‐well round bottom plates at a starting ratio of 1:40 with 100 000 T cells/well. Cells were cultured for 5 days and supernatant was harvested to measure IFN‐γ production by means of ELISA. Proliferation was assessed by the addition of 3[H]‐thymidine (0,5 μCi/well) 32. Concerning experiments with addition of exogenous fP and fH (Supporting Information Fig. 2), total CD4^+^ T‐cells were stimulated with allogenic DCs and cultured with increasing concentrations of fP or fH. The cells were cultured for 5 days after which proliferation was assessed by the addition of 3[H]‐thymidine (0,5 μCi/well). For DC characterization experiments, T cells were labeled with carboxyfluorescein diacetate succinamidyl ester (CFSE) (Molecular Probes, Europe BV Leiden, The Netherlands). In short, T cells were suspended in PBS and incubated for 15 min at 37°C with 5 μM CFSE. The reaction was quenched by washing the cells in medium containing 10% FCS before resuspending at 1 × 10^6^/mL in total RPMI culture medium. Labelled CD4^+^ T‐cells were co‐cultured with allogeneic DCs/tolDCs at a 10:1 T‐cell/DC ratio for 5 days. The T‐cells were then harvested and proliferation was assessed by CFSE dilution.

### Confocal microscopy

Cells were cultured on 8‐well chamber slides (Nunc) and stimulated overnight with IFN‐γ followed by Brefeldin‐A for the last 5 h of culture. The slides were subsequently washed and fixed in PBS containing 4% formaldehyde, 1% heat inactivated FCS followed by washing with PBS containing 1% BSA and permeabilized with perm buffer (PBS, 0.5 % saponin, 0.1% BSA) for 10 min. The DCs were then stained using purified anti‐fP or anti‐fH followed by goat anti‐mouse‐Alexa 488. Hoechst was used for nuclear staining. Stains were visualized using Leica TCS SP5 or Zeiss LSM710 NLO.

### Complement binding assays

For determination of properdin function, necrotic cells were exposed to supernatant of DC or tolDC for 1 h at 4°C, followed by detection of properdin binding by microscopy and flow cytometry. Necrosis was induced by incubating Jurkat cells at 56°C in a water bath for 1 h. And necrotic cells were confirmed by double‐staining with fluorescein isothiocyanate (FITC)–labeled annexin V and Propidium Iodide (PI)(VPS Diagnostics, Hoeven, The Netherlands) according to established methods 52. Cells were only used if a purity of >70% was achieved i.e. >70% AnnV+, PI+. The fluorescence was measured on an FACS Calibur flow cytometer, and data were analyzed with Flow Jo and Cell Quest Software. Purified human C3b 53, was coated overnight on a 96‐well Nunc plate followed by exposure to DC or tolDC supernatants and detection of properdin binding with rabbit anti human fP. To determine specificity of the assay, tolDC sups were fP depleted by incubating with anti‐properdin pre‐coupled A/G beads overnight at 4°C. Both depleted and control non‐depleted tolDC supernatant was assessed for ability to bind C3b as above. For determination of fH binding, DCs were either unstimulated or stimulated with IFN‐γ prior to supernatant exposure to coated human C3b, followed by detection by mouse anti factor H (Abcam).

### Cytokine and complement production

DCs or neutrophils were plated at 1 × 10^6^/mL in RPMI and treated as per mentioned earlier. Cell culture supernatants were harvested after indicated time points and frozen at −20°C until analysis. Subsequently they were tested for the presence of IL‐12p40 and IL‐10 by ELISA (Biolegend, Sanquin respectively) according to manufacturer's instructions, or properdin and fH using in‐house specific sandwich ELISAs 54, 55. In short, the factor H ELISA plate was coated with rabbit anti factor H and detection with rabbit anti factor H digoxigenin (DIG) followed by sheep anti‐DIG horseradish peroxidase. The properdin ELISA was performed by coating with rabbit anti‐human properdin followed by detection with rabbit anti‐human properdin DIG and sheep anti‐DIG horseradish peroxidase. For both complement protein ELISA, ABTS was used as chromogen with absorbance read at 415 nm.

Cell culture medium with 10% FCS unexposed to cells was negative in all complement assays used.

### RNA interference

DCs were transfected with 50 nM siRNA through the use of the transfection reagent Lipofectamine 2000 (Life Technologies) and were used for experiments 24 h after transfection. The following SMARTpool siRNAs were used (Dharmacon): *CFP*, *CFH* and nontargeting siRNA as a control. DC viability (PI staining) and target protein specificity were assessed (Supporting Information Fig. 1). Silencing of expression was verified by fP and fH ELISA.

### Statistical analysis

Statistical analysis was performed with Graph Pad Prism (Graph Pad Software, San Diego, CA) using a one‐tailed *t*‐test. *P*‐values ≤ 0.05 were considered statistically significant.

## Conflict of interest

The authors declare no commercial or financial conflicts of interest.

**Abbreviations**
AP: Alternative Pathway
DC: Dendritic cell
fH: Factor H
fP: properdin
tolDC: tolerogenic DC

## Supporting information

Peer review correspondence

Supplementary Figures and Tables
