# Supplementary material for: Properdin and factor H production by human dendritic cells modulates their T‐cell stimulatory capacity and is regulated by IFN‐γ
Source: Eur J Immunol. 2017 Mar 13;47(3):470–80. doi: 10.1002/eji.201646703 (PMC5363362; doi:10.1002/eji.201646703)
Supplement: Supplementary file 2 — Supplementary Figures and Tables [file EJI-47-470-s002.pdf]

European Journal of Immunology

Supporting Information
for
DOI 10.1002/eji.201646703

Karen O. Dixon, Joseph O'Flynn, Ngaisah Klar-Mohamad, Mohamed R. Daha and Cees van Kooten

Properdin and factor H production by human dendritic cells modulates their T-cell stimulatory capacity and is regulated by IFN-γ

© 2017 The Authors. *European Journal of Immunology published* by WILEY-VCH Verlag GmbH & Co. KGaA, Weinheim.

Supplementary Figures:

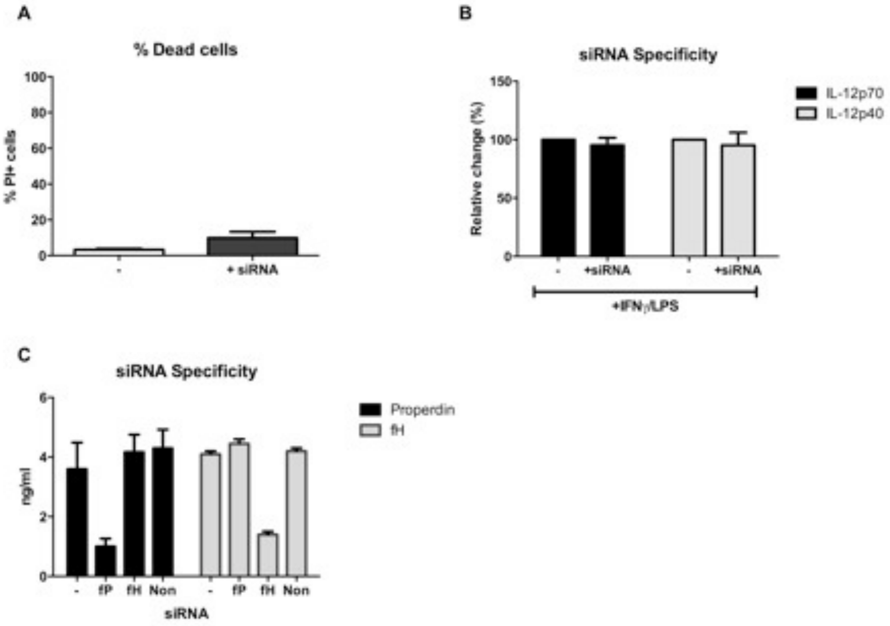

Supplementary Figure 1: DCs were either non-treated or treated with siRNA targeting fP+fH together, and assessed for cell viability (A). After 24 hours of culture, under IFNγ+LPS stimulation, supernatants were harvested, IL-12p40 and IL-12p70 was measured by ELISA (B). 100% represents the level of IL-12p40 or IL-12p70 with IFNγ+LPS stimulation but without siRNA. The level of IL-12p40/p70 in cells with IFNγ+LPS stimulation and siRNA was calculated relative to the non-siRNA treated counterparts from the same donor. DCs were either non treated, or treated with siRNA targeting fP, fH or non-specific (non) target. (C) After 24 hours of culture, supernatants were harvested and fP and fH was measured by ELISA.

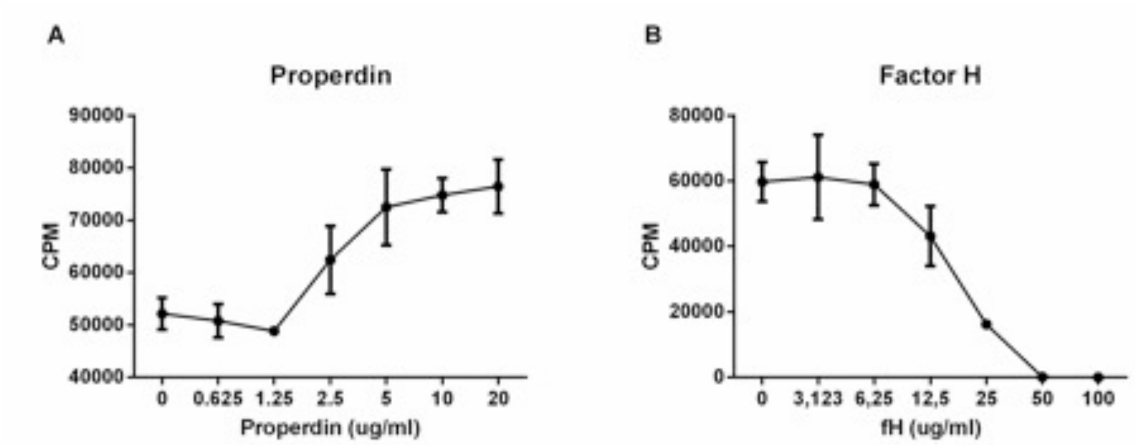

Supplementary Figure 2: Total CD4$^+$ T cells (100,000/well) were stimulated with αCD3/αCD28 coated beads for 5 days in the presence of increasing concentrations of (A) fP or (B) fH. T cell proliferation was determined at day 5 of culture by ^{3}[H] incorporation. Data shown are the mean ±SD of triplicate cultures and are representative of 2 independent experiments.

Supplementary Table 1: CT value ranges obtained in DC and tolDC for CFP and CFH under steady state (medium) or stimulated conditions (IFNγ/LPS).

DC	fP	fH	tolDC	fP	fH
Medium	23.3 - 26.4	25.0 - 30.3	Medium	22.1 – 23.6	22.5 – 24.3
IFNγ	24.7 - 27.6	23.0 - 27.8	IFNγ	23.7 – 26.5	20.4 – 23.2
LPS	23.3 - 26.3	25.1 - 30.9	LPS	20.9 – 23.7	21.8 – 25.3
