# Supplementary material for: Properdin and factor H production by human dendritic cells modulates their T‐cell stimulatory capacity and is regulated by IFN‐γ
Source: Eur J Immunol. 2017 Mar 13;47(3):470–80. doi: 10.1002/eji.201646703 (PMC5363362; doi:10.1002/eji.201646703)
Supplement: Supplementary file 1 — Peer review correspondence [file EJI-47-470-s001.pdf]

European Journal of Immunology

Properdin and factor H production by human dendritic cells modulates their T-cell stimulatory capacity and is regulated by IFN-γ

Karen O. Dixon, Joseph O'Flynn, Ngaisah Klar-Mohamad, Mohamed R. Daha, Cees van Kooten

Correspondence: Dr. Cees van Kooten, Leiden University Medical Center, Dept of Nephrology, Leiden, The Netherlands

---

Review Timeline:	Submission date:	29-Aug-2016
	First Editorial decision:	13-Oct-2016
	Revision received:	16-Dec-2016
	Second Editorial decision:	21-Dec-2016
	Accepted:	17-Jan-2017

Handling Executive Committee member: Prof. Kenneth Murphy

Please note that the correspondence below does not include the standard editorial instructions regarding preparation and submission of revised manuscripts, only the scientific revisions requested and addressed.

First Editorial Decision
13-Oct-2016

Dear Dr. Dixon,

We are sorry for a delay in the peer review of the manuscript ID eji.201646703 entitled "Properdin and factor H production by human dendritic cells is differentially regulated by IFN-γ and has a functional role in their immunogenicity." which you submitted to the European Journal of Immunology has been reviewed. There was a delay in receiving one of the reports. The comments of the referees are included at the bottom of this letter.

A revised version of your manuscript that takes into account the comments of the referee 2 will be reconsidered for publication. Should you disagree with any of the referees concerns, you should address this in your point-by-point response and provide solid scientific reasons for why you will not make the requested changes.

You should also pay close attention to the editorial comments included below. **In particular, please edit your figure legends to follow Journal standards as outlined in the editorial comments. Failure to do this will result in delays in the re-review process.**

Please note that submitting a revision of your manuscript does not guarantee eventual acceptance, and that your revision will be re-reviewed by the referees before a decision is rendered.

If the revision of the paper is expected to take more than three months, please inform the editorial office. Revisions taking longer than six months may be assessed by new referees to ensure the relevance and timeliness of the data.

Once again, thank you for submitting your manuscript to European Journal of Immunology and we look forward to receiving your revision.

Yours sincerely,

Nadja Bakocevic

On behalf of
Prof. Kenneth Murphy

Dr. Nadja Bakocevic
Editorial Office
European Journal of Immunology
e-mail: ejied@wiley.com
www.eji-journal.eu

****

Reviewer: 1

Comments to the Author
This is an excellent piece of work that provides a comprehensive evidence of a link between tolerogenic phenotype of DCs and production of factor H and properdin. The two proteins are known to have opposite functions in the complement activation. However, their local production raises a number of very important issues including their role in infection and cancer.

Reviewer: 2

Comments to the Author

1. The title is not totally clear;
2. Line 29: Dendritic cells and complement proteins participate in both innate and acquired immune response. Similar correction should be make in line 48;
3. The Introduction is missing an explanation regarding IL-27;
4. The abbreviations (such as Factor H x fH) should be consistent;
5. In the confocal microscopy analysis: what was the percentage of positive cells in each treatment and how many cells were analyzed;
6. Figure 2A and 2B: Please review conclusions regarding expression of mRNA Factor H by DC since IFN-gamma does not increase/decrease the mRNA concentration. There is no significant difference for properdin by DC or for Factor H for both DC and tolDC. In Figure 2C and 2D: how many cells were analyzed by confocal microscopy and what was the percentage of positive cells? Similar questions for Figure 5D.
7. Figure 3: How many DC, tolDC and neutrophils were used in the experiments for example in Figure 3? There is no description in Material and Methods regarding ELISA used to determine the concentration of Factor H or Properdin. Did all cell cultures supernatants have similar total protein concentrations? The legend of Figure 3 is poor;
8. Figure 5: please explain how the necrotic cells were generated;
9. Lines 275-276: please include a reference.
10. Table 1: the title is missing.
11. Supplementary Figures 1 and 2: the legends are not detailed enough.
12. Supplementary Table 1 needs a title and explanation. In my pdf copy half of the image (regarding to DC values) is black. Please check.

First Revision – authors' response

16-Dec-2016

Reviewer 2

We would like to express our appreciation for your insightful suggestions. As you will see below we have been able to revise and improve the paper as a result of your valuable feedback.

Major Points

1. **The title is not totally clear.** We would like to thank the reviewer for their comment and we apologise for any ambiguity our wording may have introduced. Our title now reads "Properdin and factor H production by human dendritic cells modulates their T cell stimulatory capacity and is regulated by IFNγ".

2. **Line 29: Dendritic cells and complement proteins participate in both innate and acquired immune response. Similar correction should be made in line 48.** We thank the reviewer for their insightful comments. We have accordingly adjusted the manuscript to reflect the important role of DCs and the complement system in not only the innate immune system but also the adaptive immune response. Changes have been made on page 3 and page 4.

3. **The introduction is missing an explanation regarding IL-27.** We have added a more thorough introduction on IL-27 in the introduction. Changes have been made on page 6.

4. **The abbreviations (such as Factor H x fH) should be consistent.** We thank the reviewer for pointing out this inconsistency in the manuscript. We have now clearly maintained the use of *CFH* to represent Factor H mRNA levels and fH to describe protein expression.

5. **In the confocal microscopy analysis: what was the percentage of positive cells in each treatment and how many cells were analyzed.**
We thank the reviewer for their comment and would like to clarify that no quantitative analysis was performed on the confocal microscopy. On average >90% of the DCs demonstrated positivity for fP and fH

6. **Figure 2A and 2B: Please review conclusions regarding expression of mRNA Factor H by DC since IFN-gamma does not increase/decrease the mRNA concentration. There is no significant difference for properdin by DC or for Factor H for both DC and tolDC. In Figure 2C and 2D: how many cells were analyzed by confocal microscopy and what was the percentage of positive cells? Similar questions for Figure 5D.** A) We have now adjusted the description of the data in Figure 2 to clearly detail the mRNA expression of *CFP* and *CFH* in DC and tolDC +/- IFNγ stimulation. Changes have been made on page 13. B) As with point 5 we would like to clarify that no quantitative analysis was performed on the confocal microscopy.
For Figure 5D we did additionally perform Flow Cytometry analysis and found that on average >95% of the necrotic cells were positive for fP binding with tolDC supernatant.

7. **Figure 3: How many DC, tolDC and neutrophils were used in the experiments for example in Figure 3? There is no description in Material and Methods regarding ELISA used to determine the concentration of Factor H or Properdin. Did all cell cultures supernatants have similar total protein concentrations? The legend of Figure 3 is poor. A)** For figure (A,D) data shown is mean ± SD of 8-24 (fP) or 4-18 (fH) independent experiments. Specifically for fP there were; DC=22, tolDC=24 and Neutrophils=8 donors, and for fH DC=17, tolDC=18 and Neutrophils=4 donors. For figures (B,C,E,F) data shown is mean ± SD of 9-28 independent experiments.

Specifically for fP in DC there were Medium=26, IFNg=26 and LPS=9 donors, and for fP tolDC=28, IFNg=28 and LPS=12 donors. For fH in DC there were Medium=17, IFNg=17 and LPS=9 donors, and for fH tolDC=22, IFNg=22 and LPS=9 donors. **B) We have now added a more thorough description of the ELISA in Material and Methods page 10. C)** As has been previously reported for other complement factors (Li, Fazekasova et al. 2011) there was donor to donor variation in the levels of fP and fH production. The overall range from the donors used in this study was fH (DC range 0.103-32.7ng/ml; tolDC 1.55-286.7ng/ml) , fP (DC 1.0-22ng/ml and tolDC 6-177ng/ml). We did not measure total protein concentrations as human monocyte derived DC do not proliferate and we plated the same number of cells for all condition analysed.

8. **Figure 5: please explain how the necrotic cells were generated.**
   Necrosis was induced by incubating Jurkat cells at 56°C in a water bath for 1 hour. and was confirmed by double-staining with fluorescein isothiocyanate (FITC)–labeled annexin V (AnnV) and Propidium Iodide (PI)(VPS Diagnostics, Hoeven, The Netherlands) according to established methods (Xu, Roos et al. 2006). Cells were only used if a purity of >70% was achieved i.e. >70% AnnV+, PI+. This has now been added to the materials and methods of the manuscript page 10.

9. **Lines 275-276: please include a reference.** We would like to thank the reviewer for highlighting this oversight. We have now included suitable references to reflect the literature on the important contribution of local DC derived complement production in regulating the allo immune response. This is now included on page 15 lines 282,283.

10. **Table 1: the title is missing.** The full title *Table 1: Real Time PCR Oligonucleotide sequences* has now been clearly added to Table I when uploading the manuscript images.

11. **Supplementary Figures 1 and 2: the legends are not detailed enough.** We would like to thank the reviewer for highlighting this. We have now included more thorough and detailed legends to each of the supplementary figures.

12. **Supplementary Table 1 needs a title and explanation. In my pdf copy half of the image (regarding to DC values) is black. Please check.**
    We apologise for this oversight which has now been rectified.

Second Editorial Decision

21-Dec-2016

Dear Dr. Dixon,

European Journal of Immunology

It is a pleasure to provisionally accept your manuscript entitled "Properdin and factor H production by human dendritic cells modulates their T cell stimulatory capacity and is regulated by IFN-γ." for publication in the European Journal of Immunology. For final acceptance, please follow the instructions below and return the requested items as soon as possible as we cannot process your manuscript further until all items listed below are dealt with.

Please note that EJI articles are now published online a few days after final acceptance (see Accepted Articles: http://onlinelibrary.wiley.com/journal/10.1002/(ISSN)1521-4141/accepted). The files used for the Accepted Articles are the final files and information supplied by you in Manuscript Central. You should therefore check that all the information (including author names) is correct as changes will NOT be permitted until the proofs stage.

We look forward to hearing from you and thank you for submitting your manuscript to the European Journal of Immunology.

Yours sincerely,
Nadja Bakocevic

on behalf of
Prof. Kenneth Murphy

Dr. Nadja Bakocevic
Editorial Office
European Journal of Immunology
e-mail: ejied@wiley.com
www.eji-journal.eu
